# Supplementary material for: “In Less than No Time”: Feasibility of Rotational Thromboelastometry to Detect Anticoagulant Drugs Activity and to Guide Reversal Therapy
Source: J Clin Med. 2022 Mar 4;11(5):1407. doi: 10.3390/jcm11051407 (PMC8911211; doi:10.3390/jcm11051407)
Supplement: Supplementary file 1 [file jcm-11-01407-s001.zip › Table_S1_rev.pdf]

Study	Type of study/ ROTEM	N. patients	Type of drug	Drug concentration or dose/ intervention	INTEM-CT	INTEM-CFT	INTEM-MCF	EXTEM-CT	EXTEM-CFT	EXTEM -MCF
Thomas [69]	In vitro	10 surgical patients	Enoxaparin vs Tinzaparin	0.5 IU/mL; 1.0 IU/m; 1.5 IU/mL<br><br>0.5 IU/mL; 1.0 IU/ml; 1.5 IU/mL	191±8 N/S; 214±109 N/S; 249±94*<br><br>223±53 N/S; 289±84 N/S; 326±125*	87±21 N/S; 85±16 N/S; 82±22§<br>74±27 N/S; 84±85 N/S; 80±21§	61±7; 63±5; 63±6<br><br>68±7; 64±8; 65±6	N/A	N/A	N/A
Feuring [72]	In vitro	16 healthy volunteers	Dalteparin vs baseline	0.01 µg/mL; 0.1 µg/mL<br><br>1 µg/mL; 10 µg/mL	126±17 N/S; 185±136 N/S<br><br>314±167°; 614±75°	74.8±17.4 N/S; 102±83 N/S<br>120±60 N/S; 251±76°	59.9±4.4 N/S; 57.8±8.0 N/S<br>55.9±9.5 N/S; 47.9±5.8°	N/A; N/A<br><br>173±112°; 332±201°	N/A; N/A;<br><br>N/A; 188±115°	N/A; N/A; N/A; 51.7±18.4
Cvirn [74]	In vitro	63 healthy volunteers	Nadropin	0 IU/mL, 0.1 IU/mL,; 0.2 IU/mL; 0.3 IU/mL; 0.4IU/mL; 0.5 IU/mL; 0.6 IU/mL; 0.7 IU/mL; 0.8 IU/mL; 0.9 IU/mL; 1 IU/mL	Dose-dependent prolongation (assay not specified)	Dose-dependent prolongation (assay not specified)	N/A	N/A	N/A	N/A
			Enoxaparin	0 IU/mL, 0.1 IU/mL; 0.2 IU/mL; 0.3 IU/mL; 0.4IU/mL; 0.5 IU/mL; 0.6 IU/mL; 0.7 IU/mL; 0.8 IU/mL; 0.9 IU/mL; 1 IU/mL	Dose-dependent prolongation (assay not specified)	Dose-dependent prolongation (assay not specified)	N/A	N/A	N/A	N/A
Jilma-Stohlawetz [75]	In vitro/ NATEM	15 healthy volunteers	Enoxaparin	0 IU/mL; 0.1 IU/mL;<br>0.2 IU/mL;<br>0.4 IU/mL	N/A; N/A;<br>prolonged by 70%<br>prolonged by 140%#	N/A; N/A;<br>prolonged by 200%<br>prolonged by 130% #	N/A	N/A	N/A	N/A
Christensen [76]	Prospective clinical	63 cancer patients	Dalteparin vs no LMWH	Preoperative<br>Perioperative<br>First postoperative day<br>Second postoperative day	165 (161-175) vs 165 (161-176) N/S<br>170 (150-173) vs 169 (162-177) N/S<br>167 (19) vs 156 (14)#<br>166 (15) vs 156 (15) N/S	N/A	N/A	58 (54-61) vs 59 (54-65) N/S<br>56 (52-58) vs 55 (53-64) N/S<br>58 (55-60) vs 59 (52-63) N/S<br>59 (55-62) vs 58 (53-67) N/S	N/A	N/A
Stanciakova [77]	Prospective clinical/ ROTEM delta	46 pregnant women	LMWH, not specified	Optimization secondary thromboprophylaxis vs controls	N/S	88 (73-111) § (10th gestation week)<br>58 (54-68)# (35th gestation week)	67 (64-70)* (26th gestation week)<br>68 (65-71)* (35th gestation week)	N/S	61 (55-67)* (26th gestation week)<br>63 (58-69)# (35th gestation week)	69 (66-72)* (26th gestation week)<br>70 (66.5-72)* (35th gestation week)
Konstantinidis [83]	Prospective clinical	60 patients undergoing carotid angioplasty	Nadroparin vs no LMWH	Dose: 5750 IU s.c. twice daily	185±63 vs 148±40§	63±17 vs 52±10 N/S	66±6 vs 66±4 N/S	N/A	N/A	N/A

Konstantinidis [83]	Prospective clinical/ Low TF-ROTEM	60 patients undergoing carotid angioplasty	Nadroparin vs no LMWH	Dose: 5750 IU s.c. twice daily	380±96 vs 293±57§	123±51 vs 86±15 §	64±10 vs 68±13 N/S	N/A	N/A	N/A
Ölander [84]	In vitro	10 ICU patients	Enoxaparin vs no LMWH	0.2 IU/mL<br>0.4 IU/mL<br>0.6 IU/mL	N/S<br>Prolonged§<br>Prolonged*	N/S<br>Prolonged#<br>Prolonged§	N/A<br>N/A<br>N/A	N/A<br>N/A<br>N/A	N/A<br>N/A<br>N/A	N/A<br>N/A<br>N/A
Ölander [84]	In vitro/ NATEM test	10 ICU patients	Enoxaparin vs no LMWH	0.2 IU/mL<br>0.4 IU/mL<br>0.6 IU/mL	N/S<br>Prolonged§<br>Prolonged°	N/S<br>N/S<br>Prolonged§	N/A<br>N/A<br>N/A	N/A<br>N/A<br>N/A	N/A<br>N/A<br>N/A	N/A<br>N/A<br>N/A
Schaden [66]	In vitro/ PiCT-ROTEM Low TF-ROTEM	16 healthy volunteers	Enoxaparin	16 LMWH concentration from 0 to 1 IU/mL<br>16 LMWH concentrations from 0 to 1 IU/mL	Linear dependency with drug concentration#<br>Linear dependency with drug concentration#	N/A<br>N/A	N/A<br>N/A	N/A<br>N/A	N/A<br>N/A	N/A<br>N/A
Eller [90]	In vitro	2 healthy volunteers	Fondaparinux	250 μg/mL (supratherapeutic)	470±59.8#	N/A	N/A	N/S	N/A	N/A
Engstrom [102]	In vitro	10 healthy volunteers	Argatroban	0 mg/L; 0.125 mg/L; 0.5 mg/L; 1 mg/L<br>2 mg/L; 4 mg/L; 8 mg/L	Strong correlation with drug concentration°	N/A	N/A	N/A	N/A	N/A
Schaden [103]	In vitro/ ECATEM	6 healthy volunteers	Argatroban	0 mg/L; 0.10 mg/L; 0.25 mg/L; 0.40 mg/L; 0.50 mg/L; 0.60 mg/L; 0.75 mg/L; 1.0 mg/L; 2.0 mg/L	N/A	N/A	N/A	ECATEM CT; strong correlation with drug concentration (r = 0.94)#	N/A	N/A
Beiderlinden [104]	Prospective clinical	17 ICU patients	Argatroban	0.5 μg/Kg/min (without liver dysfunction)<br>0.25 μg/Kg/min (with liver dysfunction)<br><br>12 hours<br>24 hours<br>48 hours<br>72 hours	↑ (compared to baseline)″<br>↑ (compared to baseline)″<br>↑ (compared to baseline)″<br>↑ (compared to baseline)″	N/A<br>N/A<br>N/A<br>N/A	N/A<br>N/A<br>N/A<br>N/A	↑ (compared to baseline)″<br>↑ (compared to baseline)″<br>↑ (compared to baseline)″<br>↑ (compared to baseline)″	N/A<br>N/A<br>N/A<br>N/A	N/A<br>N/A<br>N/A<br>N/A
Eller [90]	In vitro	19 healthy volunteers	Argatroban	0.75-2.25 μg/mL	↑ (compared to baseline) §	N/A	N/A	↑ (compared to baseline) §	N/A	N/A
Teruya [107]	Retrospective clinical	18 children on ECMO or VAD (106 blood samples)	Bivalirudin	0.33 ± 0.21 μg/kg/h continuous infusion as maintenance dose	276 ± 61 s correlated to an aPTT of 68 ± 14.8 (r = 0.54)˚; both are impacted by HLE detected in 39% of specimens; therefore, improved correlation in specimens without HLE (r = 0.68 ˚ and between HEPTEM CT (260 ± 50 s) and HPTT (61.5 ± 10.8 s) (r = 0.69)˚	N/A	N/A	109 ± 40 s correlated to a PT of 19.5 ± 2.6 s (r = 0.37)*	N/A	N/A

Legend: aPTT: activated partial thromboplastin time; CT: clotting time; CFT: clot formation time; ECATEM: ecarin-activated assay; ECMO: extracorporeal membrane oxygenation; HLE: endogenous heparin-like effects; HPTT: aPTT Hepzyme; ICU: intensive care unit; iv: intravenous; LMWH: low molecular weight heparin; MCF: maximum clot firmness; N/A = not analyzed; N/S = not significant; PiCT: prothrombinase- induced clotting time; PT: prothrombin time; ROTEM: rotational thromboelastometry; TF: tissue factor; VAD: ventricular assisted device; ↑: increased

*$p<0.001$

§$p<0.05$

°$p<0.0001$

#$p<0.01$

″$p<0.0125$
