# Supplementary material for: “In Less than No Time”: Feasibility of Rotational Thromboelastometry to Detect Anticoagulant Drugs Activity and to Guide Reversal Therapy
Source: J Clin Med. 2022 Mar 4;11(5):1407. doi: 10.3390/jcm11051407 (PMC8911211; doi:10.3390/jcm11051407)
Supplement: Supplementary file 1 [file jcm-11-01407-s001.zip › Table_S2_rev.pdf]

Study	Type of study/ ROTEM	N. patients	Type of drug	INR (Owen PT)	INTEM-CT	INTEM-CFT	INTEM-MCF	EXTEM-CT	EXTEM-CFT	EXTEM-MCF	Correlation
Schmidt [119]	Prospective clinical/ ROTEM delta	111 anticoagulated patients vs 89 healthy volunteers	Warfarin vs control	2.4 (1.9-2.9)	176 (162-191) vs 163 (157-170)*	N/A	69.4±4.1 vs 65.5±7.0 N/S	98 (83-120) vs 47 (44-51)*	N/A	68.6±4.6 vs 64.4±4.6 N/S	N/A
Schmidt [120]	Prospective clinical/ ROTEM delta	84 with VTE vs 87 healthy volunteers	Warfarin vs control	2.35 (1.9-2.73)	N/A	N/A	N/A	N/A	N/A	N/A	Lag time/EXTEM-CT (r=0.87)°
Blasi [127]	Prospective clinical/ ROTEM delta	54 consecutive patients on oral anticoagulation after elective heart valve replacement	Acenocoumarol	2.05 (1.30-2.44)	N/A	N/A	N/A	EXTEM-CT correlated at best with INR (r=0.81)°. EXTEM CT was able to predict INR values above or below 1.5: ROC AUC = 0.998. EXTEM-CT ≥ 84 s had a sensitivity and specificity of 100% and 80%, respectively, to detect an INR below 1.5. For the same INR threshold, CT ≥ 84 seconds had a predictive positive value of 92.9% and a predictive negative value of 100%	N/A	N/A	INR/EXTEM-CT (r=0.81)°
Nilsson [121]	Prospective clinical	80 anticoagulated	Warfarin	2.4 (1.9-3.4)	N/A	N/A	N/A	N/A	N/A	N/A	Owen PT/EXTEM-CT (r=0.66)° Owen PT/INTEM-CT (r=0.26) N/S Owen PT/FIBTEM-CT (r= 0.66)°
Gudmundsdottir [122]	In vitro	20 healthy donors	Warfarin	N/A	N/A	N/A	N/A	N/A	N/A	N/A	Owen PT/ROTEM-CT (r= 0.2) N/S

Legend: AF: atrial fibrillation; CT: clotting time; CFT: clot formation time; ICU: intensive care unit; INR: international normalized ratio; MCF: maximum clot firmness; N/S = not significant; N/A = not analyzed; PT: prothrombin time; ROC AUC: Receiver Operating Characteristic Area Under Curve; ROTEM: rotational thromboelastometry; VTE: venous thromboembolism

*p<0.001

°p<0.0001
