# Supplementary material for: “In Less than No Time”: Feasibility of Rotational Thromboelastometry to Detect Anticoagulant Drugs Activity and to Guide Reversal Therapy
Source: J Clin Med. 2022 Mar 4;11(5):1407. doi: 10.3390/jcm11051407 (PMC8911211; doi:10.3390/jcm11051407)
Supplement: Supplementary file 1 [file jcm-11-01407-s001.zip › Table_S3_rev.pdf]

Study	Type of study/ ROTEM	N. patients	Type of drug	INTEM-CT	INTEM-CFT	INTEM-MCF	EXTEM-CT	EXTEM-CFT	EXTEM-MCF	NATEM-CT	NATEM -CFT	NATEM-MCF	ECATEM-CT	ECATEM-CFT	ECATEM-MCF
Tsantes [138]	In vivo/ NATEM	20 NVAF vs 20 controls	Dabigatran (110 mg b.i.d)	N/A	N/A	N/A	N/A	N/A	N/A	1000±362 vs 499±116*	290±268 vs 133±32§	60.3±12.8 vs 58.8±4.0 N/S	N/A	N/A	N/A
		20 NVAF vs 20 controls	Rivaroxaban (20 mg)	N/A	N/A	N/A	N/A	N/A	N/A	916±414 vs 499±116 *	310±265 vs 133±31*	59.9±6.2 vs 58.8±4.0 N/S	N/A	N/A	N/A
Vedovati [139]	In vivo/ ROTEM gamma	10 NVAF vs 15 controls	Dabigatran (trough sample, 1 hour before after OA)	N/A	N/A	N/A	N/A	N/A	N/A	N/A	N/A	N/A	201.2±64.1 vs 99.4±14.1*	74.6±23.1 79.0±18.4 N/S	68.8±6.1 vs 64.7±4.6 §
			Dabigatran (peak sample, 2-3 hours after OA)	N/A	N/A	N/A	N/A	N/A	N/A	N/A	N/A	N/A	246.8±88.9 vs 99.4±14.1*	77.6±14.6 vs 79.0±18.4 N/S	69.1±5.5 5 vs 64.7±4.6 5§
		10 NVAF vs 15 controls	Apixaban (trough sample, 1 hour before after OA)	N/A	N/A	N/A	N/A	N/A	N/A	N/A	N/A	N/A	262.7 vs 119 vs 138±37.3#	104.2±47 vs 78±16.1§	64.5±6.6 vs 61.4±4.2 N/S
			Apixaban (peak sample, 2-3 hours after OA)	N/A	N/A	N/A	N/A	N/A	N/A	N/A	N/A	N/A	303.3±86.5 vs 138±37.3*	133.4±93.4 vs 78±16.1§	64.4±6.9 vs 61.4±4.2 N/S
		10 NVAF vs 15 controls	Rivaroxaban (trough sample, 1 hour before after OA)	N/A	N/A	N/A	N/A	N/A	N/A	N/A	N/A	N/A	243.2±36.2 vs 138±37.7*	106.6±80 vs 78±16.1 N/S	68.5±6.2 vs 61.4±4.2 N/S
			Rivaroxaban (peak sample, 2-3 hours after OA)	N/A	N/A	N/A	N/A	N/A	N/A	N/A	N/A	N/A	482.7±157.6 vs 138±37.7#	119.8±56 vs 78±16.1 N/S	66.1±9.1 vs 61.4±4.2 N/S
Korpallova [140]	In vivo/ ROTEM gamma	45 NVAF vs controls	Rivaroxaban (20 mg) (trough sample)	164.5±4.8 vs 53.4±5.8°	59±4.6 vs 84.1±4.3§	66.2±1.7 vs 60.9±3.7 N/S	101.9±8.3 vs 55.3±6.5°	66±7.2 vs 80±23.8 N/S	68.4±1.8 vs 63.5±4.2§	N/A	N/A	N/A	N/A	N/A	N/A
			Rivaroxaban (20 mg) (peak	221.3±6.4 vs 53.4±5.8°	58.8±3.5 vs 84.1 ±4.3#	66.9 ±1.7 vs 60.9±3	191.8±22.9 vs	68.3±2.7 vs 80±23.8 N/S	68.8±1.3 vs 63.5±4.2#	N/A	N/A	N/A	N/A	N/A	N/A

			sample)			N/S	55.3±6.5°								
Korpallova [140]	In vivo/ ROTEM gamma	45 NVAF vs controls	Apixaban (5 mg b.i.d) (trough sample)	176.6±4.9 vs 53.4±5.8°	66.9 ±3.3 vs 84.1±4.3 N/S	63.6±1.1 vs 60.9±3.7 N/S	103±4.9 vs 55.3±6.5 °	68.4±3 vs 80±23.8 N/S	66.6±1.1 vs 63.5±4.2 N/S	N/A	N/A	N/A	N/A	N/A	N/A
			Apixaban (5 mg b.i.d) (peak sample)	193.2±9.1 vs 53.4±5.8°	65.1±1.4 vs 84.1±4.3 N/S	65.4±1.4 vs 60.9±3.7 N/S	69.9±5.1 vs 80±23.8 N/S	69.9±5.1 vs 80±23.3 N/S	68± 1.3 vs 63.5±4.2§	N/A	N/A	N/A	N/A	N/A	N/A
Taune [143]	Ex vivo	30 NVAF	Dabigatran (150 mg b.i.d)	Correlation with plasma concentration (r=0.73)#	N/A	N/A	Correlation with plasma concentration (r=0.92)#	N/A	N/A	N/A	N/A	N/A	N/A	N/A	N/A
Herrmann [144]	In vivo	17 NVAF	Dabigatran (150 mg b.i.d)	Correlation with plasma concentration (r=0.679)#	N/A	N/A	Correlation with plasma concentration (r=0.838)°	N/A	N/A	N/A	N/A	N/A	N/A	N/A	N/A
Herrmann [144]	In vivo	15 NVAF	Rivaroxaban (10 mg)	Correlation with plasma concentration (r=0.128) N/S	N/A	N/S	Correlation with plasma concentration (r=0.164) N/S	N/A	N/A	N/A	N/A	N/A	N/A	N/A	N/A
Klages [145]	Ex vivo	16 NVAF/ VTE	Dabigatran	Correlation with plasma concentration (r=0.792)°	Correlation with plasma concentration (r=0.051) N/S	Correlation with plasma concentration (r=0.192) N/S	Correlation with plasma concentration (r=0.765)°	Correlation with plasma concentration (r=-0.083) N/S	Correlation with plasma concentration (r= 0.354)§	N/A	N/A	N/A	N/A	N/A	N/A
		16 NVAF/VTE	Rivaroxaban	Correlation with plasma concentration (r=0.689)°	Correlation with plasma concentration (r=0.022) N/S	Correlation with plasma concentration (r=-0.063) N/S	Correlation with plasma concentration (r=0.595)*	Correlation with plasma concentration (r=-0.008) N/S	Correlation with plasma concentration (r=-0.013) N/S	N/A	N/A	N/A	N/A	N/A	N/A
Comuth [146]	Ex vivo	15 NVAF	Dabigatran (8 patients 150 mg b.i.d and 7 patients	Correlation with plasma concentration (R^2= 0.92)#	N/A	Correlation with plasma concentra	Correlation with plasma concentrati	N/A	Correlation with plasma concentration (R^2= 0.01) N/S	N/A	N/A	N/A	N/A	N/A	N/A

	In vitro	10 healthy volunteers	110 mg b.i.d)<br><br>Dabigatran	Correlation with plasma concentration (R^2=0.98)°	N/A	tion (R^2= 0.32) N/S<br><br>Correlation with plasma concentration (R^2= 0.75)#	on (R^2=0.95) °<br><br>Correlation with plasma concentration (R^2= 0.97)°	N/A	Correlation with plasma concentration (R^2= 0.75)#	N/A	N/A	N/A	N/A	N/A	N/A
Sokol [147]	In vivo/ ROTEM gamma	27 NVAF	Dabigatran (150 mg b.i.d, before vs 2 hours after OA)	Correlation with plasma concentration (r=0.84)°	Correlation with plasma concentration (r=0.23) N/S	Correlation with plasma concentration (r=-0.92)°	Correlation with plasma concentration (r=0.92)°	Correlation with plasma concentration (r=0.89)°	Correlation with plasma concentration (r= -0.93)°	N/A	N/A	N/A	N/A	N/A	N/A
Korber [148]	Ex vivo/ ECATEM	10 hip and knee arthroplasty	Dabigatran (110 mg b.i.d.)	Correlation with plasma concentration 2 hours after OA (r= 0.88)*<br><br>Correlation with plasma concentration 6 hours after OA (r= 0.53) N/S<br><br>Correlation with plasma concentration 12 hours after OA (r= 0.83)#	N/A	N/A	Correlation with plasma concentration 2 hours after OA (r= 0.69) N/S<br><br>Correlation with plasma concentration 6 hours after OA (r= 0.54) N/S<br><br>Correlation with plasma concentration 12 hours after OA(r= 0.50) N/S	N/A	N/A	N/A	N/A	N/A	Correlation with plasma concentration 2 hours after OA (r= 0.90)*<br><br>Correlation with plasma concentration 6 hours after OA (r= 0.77)#<br><br>Correlation with plasma concentration 12 hours after OA (r= 0.85)#	N/A	N/A

Schäfer [149]	In vitro/ ROTEM delta-ECATEM	10 healthy volunteers	Dabigatran	N/A	N/A	N/A	N/A	N/A	N/A	N/A	N/A	N/A	High correlation with spiked blood concentrations (r=0.9985)*	N/A	N/A
			Rivaroxaban	N/A	N/A	N/A	N/A	N/A	N/A	N/A	N/A	N/A	N/S	N/A	N/A
Seyve [151]	In vitro/ ROTEM delta	20 healthy volunteers	Apixaban (n. 5 patients)	>1200 ng/mL for double	N/A	N/S	1042±225 ng/mL for double	N/A	N/S	N/A	N/A	N/A	N/A	N/A	N/A
			Edoxaban (n. 5 patients)	715±134 ng/mL for double	N/A	N/S	134±38 ng/mL for double	N/A	N/S	N/A	N/A	N/A	N/A	N/A	N/A
			Rivaroxaban (n. 5 patients)	1088±114 ng/mL for double	N/A	N/S	176±26 ng/mL for double	N/A	N/S	N/A	N/A	N/A	N/A	N/A	N/A
			Dabigatran (n. 5 patients)	449±94 ng/mL for double	N/A	N/S	284±73 ng/mL for double	N/A	N/S	N/A	N/A	N/A	N/A	N/A	N/A
Mahamad [152]	In vivo/ ROTEM delta	12 bleeding patients	Dabigatran (n.1 patient)	N/A	N/A	N/A	N/A	N/A	N/A	N/A	N/A	N/A	N/A	N/A	N/A
			Rivaroxaban (n.3 patients)	N/A	N/A	N/A	Correlation with PT value (r=0.86) N/S	N/A	N/A	N/A	N/A	N/A	N/A	N/A	N/A
			Apixaban (n.8 patients)	N/A	N/A	N/A	Correlation with PT value (r=0.56) N//S	N/A	N/A	N/A	N/A	N/A	N/A	N/A	N/A
Kyriakou [156]	In vivo/ NATEM	40 NVAF vs 20 controls	Rivaroxaban (20 mg) (n. 20 patients)	N/A	N/A	N/A	N/A	N/A	N/A	916±414 vs 499±116*	310±265 vs 133±31*	59.9±6.2 vs 58.8±4 N/S	N/A	N/A	N/A
			Apixaban (5 mg b.i.d) (n. 20 patients)	N/A	N/A	N/A	N/A	N/A	N/A	750±150 vs 499±116*	232±128 vs 133±31#	60.2±7.6 vs 58.8±4 N/S	N/A	N/A	N/A

Schenk [157]	Ex vivo/ ROTEM gamma	13 healthy volunteers	Rivaroxaban (15 mg or 20 mg, before vs 3 hours after OA)	Correlation with PT (r=-0.740)§	N/A	N/A	68±6 vs 425±146§ Correlation with PT (r=-0747)§	N/A	N/A	N/A	N/A	N/A	N/A	N/A	N/A
Casutt [158]	Ex vivo	11 healthy volunteers	Rivaroxaban (10 mg, before vs 2.5 hours after OA)	194±26 vs 239±43§	86±11 vs 87±16 N/S	56±2 vs 54±60 N/S	58±9 vs 87±17#	113±30 vs 87±20 #	57±5 vs 57±4 N/S	N/A	N/A	N/A	N/A	N/A	N/A
Chojnowski [159]	In vivo	13 VTE patients	Rivaroxaban (20 mg, before vs 2.5 hours after OA)	200.5±58.1 vs 272.1±133.8*	N/A	N/A	77.2±45.4 vs 178.5±118.5*	N/A	N/A	N/A	N/A	N/A	N/A	N/A	N/A
Fontana [163]	Ex-vivo/ ROTEM delta	20 healthy volunteers	Rivaroxaban (20 mg, before vs 3 hours after OA)	56.1 (41.8, 70.3)°	2.8 (-3.2, 8.7) N/S	-0.5 (1.3, 0.4) N/S	47.5 (37.8,-57.1)°	-17.3 (-22.8,-11.7)°	2.2 (1.3, 3.1)*	N/A	N/A	N/A	N/A	N/A	N/A
Havrdova [164]	In vitro/ ROTEM delta	15 healthy volunteers	Edoxaban (60 mg)	N/A	N/A	N/A	Correlation with plasma concentration 2 hours (r=0.90)*, 4 hours (r=0.84)*, 6 hours (r= 0.77)*, 8 hours after OA (r= 0.52)§	N/A	N/S	N/A	N/A	N/A	N/A	N/A	N/A

Legend: OA: oral administration; b.i.d= bis in die; CFT: clot formation time; CT: clotting time; ECATEM: ecarin-activated assay; ICU: intensive care unit; LMWH: low molecular weight heparin; MCF: maximum clot formation; N/A = not analyzed; NATEM: nonactivated thromboelastometry; N/S = not significant; NVAF: non valvular atrial fibrillation; PT: prothrombin time; PiCT: prothrombinase-induced clotting time; ROTEM = rotational thromboelastometry; TF: tissue factor; VTE: venous thromboembolism

*p<0.001

§p<0.05

°p<0.0001

#p<0.01
